# Monitoring Antiseizure Medication Change Using Ultra Long‐Term Electroencephalogram: A Multicenter Study

**DOI:** 10.1002/ana.78305

**Published:** 2026-07-20

**Authors:** Pedro F. Viana, Matthew McWilliam, Johannes Koren, Christina Duarte, Tamara Lisy, Christoph Baumgartner, Karl Rössler, Matthias Tomschik, Adriana Celdrán de Castro, Rodrigo Rocamora, Laura Vilella, Guido Rubboli, Sigge Weisdorf, Cecilia Friedrichs‐Maeder, Troels W. Kjaer, Sanchita Bhatia, Dympna McAleer, Khalid Hamandi, Deborah Spencer, Rajiv Mohanraj, Mark P. Richardson

**Affiliations:** ^1^ Institute of Psychiatry, Psychology and Neuroscience, King's College London London United Kingdom; ^2^ Center for Epilepsy, King's College Hospital NHS Foundation Trust London United Kingdom; ^3^ Department of Neurology Clinic Hietzing Vienna Austria; ^4^ Karl Landsteiner Institute for Clinical Epilepsy Research and Cognitive Neurology Vienna Austria; ^5^ Department of Neurosurgery Medical University of Vienna Vienna Austria; ^6^ Epilepsy Center, Hospital del Mar Research Institute (IMIM) Barcelona Spain; ^7^ Universitat Pompeu Fabra (UPF) Barcelona Spain; ^8^ Danish Epilepsy Center Dianalund, Denmark; ^9^ Institute of Clinical Medicine University of Copenhagen Copenhagen Denmark; ^10^ Sleep–Wake–Epilepsy Center, NeuroTec, Center for Experimental Neurology, Department of Neurology, University Hospital Bern, Inselspital, University of Bern Bern Switzerland; ^11^ Department of Health Science and Technology University of Aalborg Aalborg Denmark; ^12^ The Welsh Epilepsy Centre, Department of Neurology University Hospital of Wales Cardiff United Kingdom; ^13^ Department of Neurology Manchester Centre for Clinical Neurosciences, Northern Care Alliance NHS Foundation Trust Manchester United Kingdom; ^14^ Division of Neuroscience Faculty of Biology Medicine and Health, University of Manchester Manchester United Kingdom

## Abstract

**Objective:**

Medication dose adjustments are common in treatment‐resistant epilepsy, but lack robust evidence, and patient‐reported seizure diaries have known limitations. We assessed whether ultra long‐term subcutaneous electroencephalogram (sqEEG) improves detection of seizure frequency changes after antiseizure medication adjustments.

**Methods:**

A retrospective, multicenter cohort study was carried out across 9 centers in adults with treatment‐resistant epilepsy. SqEEG seizure frequency was compared with seizure diaries before and after medication dose increases and reductions, over 7‐, 14‐, and 30‐day windows, using categorical analyses, mixed‐effects models and survival analyses.

**Results:**

A total of 65 participants (mean age 40.6 years; 59% female) contributed >280,000 hours of sqEEG, 9,190 electrographic seizures and 4,850 diary events, including across 160 medication changes. Categorical disagreement between modalities ranged from 36% to 50%, with significant differences at 7 and 14 days for dose reductions, and at 14 days for dose increases. sqEEG recorded 2.2‐ to 3.4‐fold more seizures than diary across all seizure count models (all p < 0.01). Seizure count models showed no statistically significant difference between modalities in detecting treatment‐related changes (interaction estimates: 5–38% larger increases with sqEEG for dose reductions, and 13–38% larger reductions with sqEEG for dose increases). SqEEG identified return to baseline seizure counts significantly earlier than diary after dose reductions at 7‐ and 14‐day windows (median 7 vs 13 days and 10 vs 17 days, respectively; adjusted p = 0.046 and 0.042).

**Interpretation:**

SqEEG detects more seizures than patient‐reported diaries and identifies treatment‐related changes earlier, even after modest medication adjustments, particularly after dose reductions, highlighting the potential of objective ultra long‐term EEG monitoring in treatment‐resistant epilepsy. ANN NEUROL 2026;100:600–612

Despite the ongoing introduction of new antiseizure medications (ASMs), the prevalence of treatment‐resistant epilepsy remains largely unchanged, with many individuals continuing to experience recurrent seizures.^1^ Seizures are potentially catastrophic events, associated with injury, psychosocial harm, and increased mortality.^2^, ^3^, ^4^

In routine clinical practice, epilepsy treatment commonly involves repeated medication adjustments over time. However, apart from simulation studies,^5^ there is limited evidence to support specific dosing strategies in real‐world clinical settings, and treatment decisions are often guided by a trial‐and‐error approach. A commonly adopted strategy is to “start low and go slow,” titrating ASMs until a maximally tolerated or presumed effective dose is reached.^6^ A major limitation of these approaches is the lack of a rapid and reliable method to assess changes in seizure frequency or disease control after treatment modifications. This uncertainty can lead to prolonged periods of ineffective treatment, increased healthcare costs, and ongoing morbidity for people with epilepsy.

Seizure frequency is typically assessed using patient‐reported seizure diaries, in routine clinical settings, as well as in clinical trials of ASMs. However, multiple studies have demonstrated that seizures are frequently misdocumented or underreported in patient‐reported diaries.^7^ It remains unclear whether seizure misdocumentation primarily occurs in a linear fashion (such that reported seizures follow similar temporal trends, but systematically underestimate true seizure counts) or whether reporting accuracy varies substantially over time. One study comparing long‐term intracranial EEG monitoring with patient diaries suggested the latter.^8^

Reporting accuracy might fluctuate for several reasons. These include placebo effects, such as perceived improvement after treatment initiation or reassurance during medication tapering despite ongoing seizures, as well as cognitive and memory effects of seizures and ASMs. In addition, seizure semiology might change, and seizures might become more or less perceivable after ASM adjustments.

Against this background, there has been rapid development of novel seizure monitoring systems designed to reliably detect and quantify seizures from home.^9^, ^10^ Among these, mobile electroencephalogram (EEG), such as subcutaneous EEG (sqEEG), is a promising modality, as it directly measures electrographic brain activity, and enables the detection of a wide variety of seizure patterns and types.^11^ Recent studies have shown that sqEEG monitoring is safe, feasible, and provides stable, highly‐quality signal over months to years (ultra long term).^11^, ^12^, ^13^, ^14^, ^15^, ^16^ Emerging case reports suggest an added benefit of sqEEG during periods of treatment change, including the detection of unreported breakthrough seizures.^15^, ^17^ In contrast sqEEG might also detect electrographic seizures without overt clinical correlates, which might occur independently of medication changes and have limited direct impact on patients' daily lives.

Seizure frequency shows natural variability, including cyclic variability^18^ and seizure frequency‐dependent variability,^19^ making it challenging to accurately assess the individual efficacy of a change in antiseizure medication. In this study, we pooled data from individuals undergoing ultra long‐term subcutaneous EEG monitoring to objectively quantify seizures around periods of ASM adjustment, and compared these measurements with simultaneous seizure diary reports. We hypothesized that objective seizure monitoring using sqEEG would provide added benefit in capturing medication‐related changes in seizure frequency, thereby enhancing the clinical utility of ambulatory objective seizure monitoring in both research and routine clinical practice.

## Methods

### Study Design, Population, and Procedures

**Figure 1:**
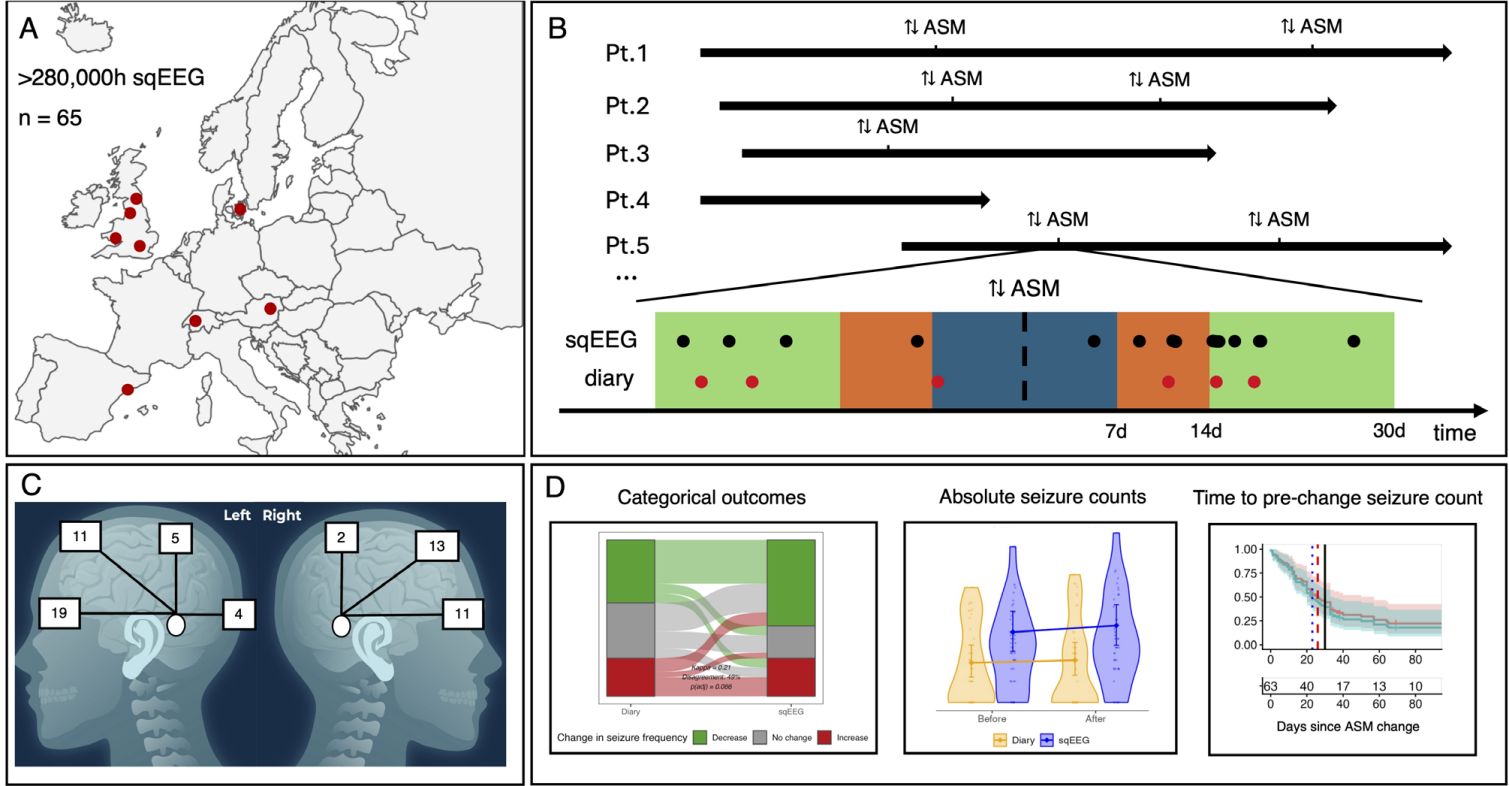
Study design and population. (A) Geographic distribution of centers contributing longitudinal subcutaneous electroencephalogram (sqEEG) data. (B) Schematic of study design and observation window stratification. (C) Schematic of subcutaneous electroencephalogram implant locations and their distribution in the included cohort. (D) Overview of the 3 analytical approaches. ASM = antiseizure medication. [Color figure can be viewed at www.annalsofneurology.org]

We conducted a retrospective observational multicenter study. We analyzed data from adult participants with treatment‐resistant epilepsy who underwent subcutaneous EEG implantation and recordings from different studies in 9 centers in Austria, Denmark, Spain, Switzerland, and the UK (Fig. 1A). Each contributing study had received approval from its respective institutional ethics review board, including approval to share anonymized data. Inclusion criteria varied across studies, but overall consisted of adult patients with treatment‐resistant epilepsy, with seizures detectable in 2‐channel EEG, and without contraindications to device implantation.^20^

Patients were monitored using the 24/7 EEG™ SubQ system (UNEEG medical). The UNEEG SubQ™ implant consists of a 3‐contact lead providing 2 bipolar EEG channels and a small ceramic housing implanted unilaterally, typically under local anesthesia over regions of known or suspected ictal activity. Recording typically began 1–4 weeks after implantation. An external recorder powers the implant via an inductive link and stores the data. Signals are recorded at a sampling rate of 207 Hz. Participants were asked to use the system as continuously as possible during daily life and report seizures in a diary, either electronic or in paper format. Changes to habitual ASM were allowed as per clinical indication throughout the recordings.

### Data Collected

We collected demographic and epilepsy‐related clinical data, patient‐ or caregiver‐reported seizure diary entries, and sqEEG recording information. SqEEG data included total recording duration, participant‐specific recording adherence, and timestamps of annotated sqEEG seizures. The sqEEG data were annotated by each center's reporting team(s), which included board‐certified neurophysiologists. Each center provided sqEEG seizure timestamps after expert human annotator review of the data, with varying approaches to including additional information to assist the reviewer. Although the annotation strategy varied between centers, all strategies were aimed at high sensitivity. Centers used 1 of 3 broad approaches to annotation: (1) expert review of high‐sensitivity annotations generated by a seizure‐detection algorithm (Episight)^21^; (2) expert review of the full datasets without additional automated assistance (eg, as reported by Rubboli et al.^14^); or (3) a combination of the 2 (eg, as reported by Viana et al.^12^). A previous study reported a sensitivity of expert visual review of full sqEEG against gold standard video‐EEG to be 100%, and sensitivity of expert review of EpiSight against video‐EEG review of 67%.^14^ In the ambulatory setting, across studies, EpiSight review showed a median per‐patient sensitivity of 70.5 to 91.5% against full visual sqEEG review.^12^, ^14^, ^21^ Data from 3 previously published studies are included in this cohort (see Supplementary Material 1 for more details on each center's annotation strategy. No center relied on automated EpiSight detections alone).^12, 14, 21^

Antiseizure medication changes were annotated throughout the recording period. Time periods surrounding routine daily ASM changes were analyzed (Fig. 1B). We included treatment adjustments involving changes in the prescribed daily dose of an ASM, categorized as either medication increases (dose escalation of an existing ASM or initiation of a new ASM) or medication reductions (dose reduction of an existing ASM or discontinuation of an ASM). Individual medication doses were converted to defined daily doses according to the definitions of the World Health Organization Collaborating Center for Drug Statistics Methodology.^22^ Total drug load was calculated as the sum of defined daily doses across all concurrently prescribed antiseizure medications at each timepoint. Administration of rescue ASMs or missed medication doses were not analyzed. The use of sqEEG data in clinical decision‐making varied across centers, ranging from strictly observational to periodic review with patients and clinicians. Real‐time feedback at weekly or biweekly intervals was not standard practice at any center; where clinical review occurred, it was typically at 30‐day intervals or longer.

### Statistical Analysis

We first aimed to characterize statistical properties of seizure frequency data for both modalities. The time‐of‐day distribution of sqEEG and diary seizure timestamps was compared after excluding calendar‐date‐only diary entries, where no time of day was recorded (Supplementary Material 2). The log–log relationship between within‐patient mean and standard deviation of binned seizure counts was examined for both modalities across all window lengths to characterize the statistical distribution of seizure counts and inform model selection (Supplementary Material 3).^19^ Per‐patient Spearman correlations between binned sqEEG and diary seizure counts were computed for 7‐, 14‐, and 30‐day bin lengths to assess within‐individual concordance between modalities over time (Supplementary Material 4).^8^ Calibration plots of diary versus sqEEG seizure counts (pre, post, and post minus pre, log‐transformed) were constructed to assess offset and/or scaling bias (Supplementary Material 5).

Seizure frequency was compared between time windows before and after routine daily ASM changes. Pre‐ and post‐change observation windows of 7, 14, and 30 days were defined empirically to reflect timeframes that could feasibly be adopted in routine clinical review of sqEEG data, while balancing treatment latency effects and data availability. Early (1–2 weeks) detection rate changes from responsive neurostimulation have been shown to predict the efficacy of newly added ASMs.^23^ The number of eligible medication‐change trials decreased substantially with increasing window length (Supplementary Material 6). In the primary analysis, trials were included if a complete recording time was present for the full duration of both observation windows, regardless of the presence of other concurrent ASM change. A sensitivity analysis was then performed, restricting analyses to nonoverlapping trials, defined as those with no additional ASM changes occurring within the pre‐ or post‐change windows. Analyses were complete‐case with respect to observation window availability; no imputation of missing seizure counts was performed. SqEEG recording adherence was not used as an inclusion criterion, as trials were retained regardless of adherence level.

Analyses were conducted separately for ASM dose increases and dose decreases, and across 3 complementary approaches (Fig. 1D).

First, seizure frequency changes were categorized as a decrease, no change, or increase after medication adjustment, based on the absolute difference in seizure count between post‐ and pre‐change windows (positive difference = increase, negative = decrease, zero = no change). These categorical outcomes were paired between seizure diary and sqEEG for each trial. Differences in the distribution of paired categorical outcomes between modalities were assessed using the Stuart–Maxwell test for marginal homogeneity. Separate analyses were conducted for each observation window and ASM change category. To quantify agreement beyond chance, linearly weighted Cohen's kappa was computed for each window and change type, treating the 3 categories as ordered. To assess whether categorical comparisons were sensitive to the exact‐zero definition of “no change,” a sensitivity analysis was conducted in which the categorical analysis was recomputed across tolerance bands of ±*k* seizures (*k* = 0 to 5), with *k* = 5 corresponding to the upper interquartile range of the seizure‐count change distribution for sqEEG (Supplementary Material 7).

Second, absolute seizure counts derived from seizure diaries and sqEEG were compared. Seizure count data were highly overdispersed and with a large proportion of zero counts (Supplementary Material 8), and previous work has suggested that a negative binomial distribution better approximates seizure frequency.^24^ Accordingly, negative binomial generalized linear mixed‐effects models were fitted with seizure count as the outcome, subject identifier as a random intercept, and fixed effects for modality (diary vs sqEEG), time (pre‐ vs post‐change), and their interaction. The primary outcome of interest was modality × time interaction (assessing whether 1 modality detected larger treatment‐related changes in seizure frequency), and a secondary outcome was the main effect of modality (assessing whether 1 modality systematically detected more seizures). Model fit was evaluated using simulation‐based scaled residual diagnostics from the DHARMa package (1,000 simulations), including tests for overdispersion, zero inflation, and residual uniformity. Quantile–quantile plots were inspected to visually confirm model fit. To assess whether seizure cyclicity could systematically bias pre‐ to post‐change comparisons at the group level, we computed per‐patient autocorrelation functions of daily sqEEG and diary seizure counts across lags from 5 days to one‐quarter of each patient's recording length (capped at 90 days – see Supplementary Material 9).

Third, we assessed whether either modality detected the effects of ASM changes earlier than the other using survival analysis. The outcome was time to return to the modality‐specific pre‐change seizure count following a medication adjustment, conceptually analogous to the “time to pre‐randomization” endpoint used in clinical trials.^25^ We note that the conventional “time to pre‐randomization” endpoint typically refers to the time at which the seizure count reaches or exceeds that observed over the average month in a baseline phase (often 8–12 weeks); whereas in this analysis, we used the absolute pre‐change seizure count over a matched observation window (7, 14, or 30 days) on each modality's own scale. Time‐to‐seizure count designs with short time windows (7–28 days) have been proposed and tested in pediatric focal epilepsy clinical trial data.^26^, ^27^ Cox proportional hazards models were fitted with modality as a fixed‐effect covariate, and robust standard errors clustered by subject to account for repeated medication changes within individuals.

For all analyses, statistical significance was set at *p* < 0.05 with adjustment for multiple comparisons using the Benjamini–Hochberg procedure applied globally across all 3 analytical approaches (categorical outcomes, seizure count models, and survival analyses), treatment change types, and observation windows. Data processing and curation were performed using Python (v3.7.3), and statistical analyses were conducted in RStudio (v2025.09.2) including the glmmTMB, DHARMa, emmeans, DescTools, irr, and survival packages.

## Results

### Cohort Description

A total of 65 participants comprised the study cohort (Table 1). The mean age was 40.6 years (SD 12.3), and 59% were women. A total of 64 participants had focal epilepsy, with the majority (*n* = 33, 50.8%) having an unknown etiology. One patient had generalized epilepsy. Focal impaired consciousness seizures were the most reported seizure type (*n* = 58, 89.2%), and 38.5% of the participants also reported tonic–clonic seizures.

**Table 1:** Cohort Demographic, Clinical, and Recording Characteristics

	sqEEG cohort (*n* = 65)
Age at the start of the recording, mean (SD)	40.60 (12.33)
Female sex, *n* (%)	38 (58.5%)
Epilepsy type and etiology, *n* (%)	
Generalized genetic	1 (1.5%)
Focal immune	8 (12.3%)
Focal infectious	2 (3.1%)
Focal structural	21 (32.3%)
Focal unknown	33 (50.8%)
Seizure types reported per patient, *n* (%)	
Generalized tonic–clonic	1 (1.5%)
Other generalized	1 (1.5%)
Focal preserved consciousness	26 (40.0%)
Focal impaired consciousness	58 (89.2%)
Focal to bilateral tonic–clonic	38 (58.5%)
No. ASM at baseline, *n* (%)	
0	2 (3.1%)
1	4 (6.2%)
2	18 (27.7%)
3	25 (38.5%)
4	14 (21.5%)
5	2 (3.1%)
Total no. diary‐reported events (*n*)	4,850
Average diary reporting frequency per day, mean (SD)	0.41 (0.89)
Total no. sqEEG recorded seizures	9,190
Average sqEEG seizure frequency per day, mean (SD)	0.81 (2.38)
sqEEG recording duration (days), median (IQR)	190 (92–317)
sqEEG recording percentage adherence, median (IQR)	81.4% (67.8–91.7%)
Antiseizure medication changes (*n*)	160
ASM increase trials, *n* (%)	101 (63.2%)
Change in total DDD drug load, median (IQR)	6% (2–12%)
ASM reduction trials, *n* (%)	59 (36.9%)
Change in total DDD drug load, median (IQR)	5% (6–10%)

Abbreviations: ASM = antiseizure medication; DDD = defined daily dose; IQR = interquartile range; sqEEG = subcutaneous electroencephalogram.

Participants underwent sqEEG monitoring with concurrent seizure diary reporting for a median duration of 190 days or 6.3 months (interquartile range [IQR] 92–317 days, or 3–10.4 months). Median sqEEG recording adherence was 81.4% (IQR 67.8–91.7%), corresponding to approximately 19.5 h (16.3–22.0 h) of recording per day. Most sqEEG implantations were positioned over frontotemporal regions, with a predominance of left‐hemispheric placements (Fig. 1C).

At study baseline, most participants were prescribed 2 (*n* = 18, 27.7%), 3 (*n* = 25, 38.5%), or 4 (*n* = 14, 21.5%) ASMs. A total of 160 ASM change trials meeting complete observation window criteria for the primary analysis were identified. Medication increases comprised dose adjustments of existing ASMs (*n* = 83) and initiation of a new ASM (*n* = 18), whereas medication reductions comprised dose adjustments of existing ASMs (*n* = 40) and discontinuation of an existing ASM (*n* = 19). The distribution of number of ASM trials per patient is found in Supplementary Material 10. In patients with >1 medication change, the median time between changes was 16 (IQR 13.8–35) days. The most common treatments increased were cenobamate, lamotrigine, and brivaracetam, whereas the most frequent treatment reductions were lacosamide, clobazam, and lamotrigine (Supplementary Material 11).

Medication increases were mostly small in magnitude, with a median increase in total defined daily doses‐defined drug load of 6% (IQR 3–10%). Medication reductions were similarly modest, with a median decrease in total drug load of 6% (IQR 2–12%).

### Statistical Distribution and Correlation of Seizure Counts

The time‐of‐day distribution of seizure timestamps differed markedly between modalities (Supplementary Material 2). sqEEG seizures were distributed across the full 24‐hour period, whereas diary‐reported seizures were predominantly clustered during waking hours. Both sqEEG and diary seizure counts followed a log–log relationship between within‐patient mean and standard deviation across all window lengths (Supplementary Material 3). Per‐patient Spearman correlations between binned sqEEG and diary seizure counts showed low‐to‐moderate agreement across all window lengths, with median r values of 0.45 (IQR 0.16–0.66), 0.39 (IQR 0.19–0.65), and 0.53 (IQR 0.24–0.71) for 7‐, 14‐, and 30‐day bins, respectively, with wide IQRs reflecting substantial between‐patient variability in concordance (Supplementary Material 4). Calibration plots comparing diary and sqEEG seizure counts (pre‐change, post‐change, and post minus pre‐change, log‐transformed) are presented in Supplementary Material 5, showing a consistent offset bias, with sqEEG detecting seizures when diary counts were zero, and weak positive correlation between modalities (*R* ^2^ = 0.11–0.40).

### Categorical Outcomes

**Figure 2:**
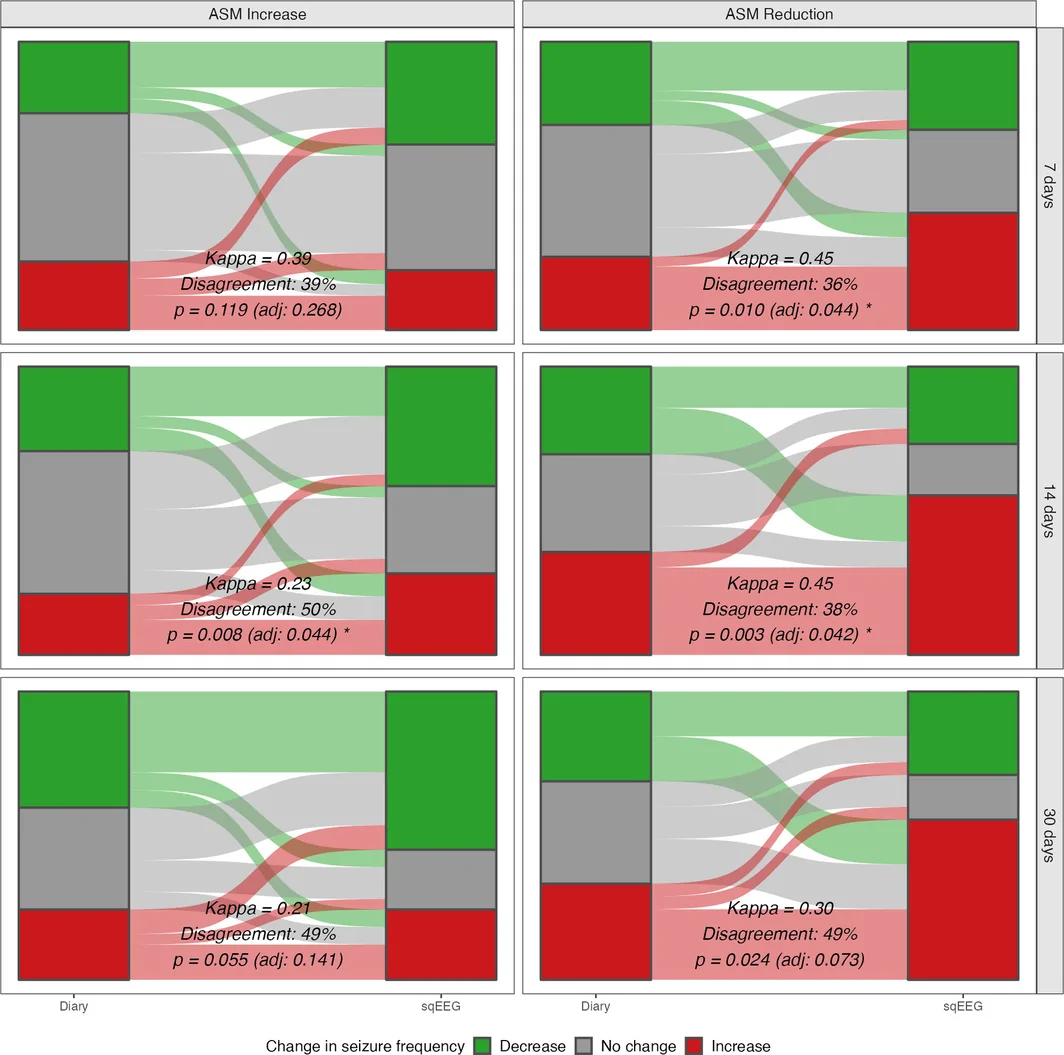
Flow of seizure frequency classifications between patient‐reported diary and subcutaneous electroencephalogram across treatment change categories and observation windows. Each Sankey diagram displays the proportion of trials flowing from diary classification (left) to subcutaneous electroencephalogram classification (right), for antiseizure medication increase and antiseizure medication reduction trials separately, across 7‐, 14‐, and 30‐day observation windows. Green indicates seizure frequency decrease (improvement), gray indicates no change, and red indicates seizure frequency increase (worsening). Ribbon width is proportional to the fraction of trials. Weighted Cohen's kappa, overall proportion of discordant classifications, and Stuart–Maxwell adjusted p‐values are displayed within each panel. *Adjusted *p* < 0.05. ASM = antiseizure medication [Color figure can be viewed at www.annalsofneurology.org]

We assessed whether seizure diaries and sqEEG differed in their classification of seizure frequency changes using a simple categorical framework aligned with clinical practice (decrease, no change, or increase in seizure frequency). Paired Stuart–Maxwell tests demonstrated significant discrepancies between modalities for ASM dose increases at the 14‐day window (adjusted *p* = 0.044), and for ASM dose reductions at the 7‐ and 14‐day windows (adjusted *p* = 0.044 and 0.042, respectively; Fig. 2 and Supplementary Material 12). Weighted kappa values ranged from 0.21 to 0.45, indicating fair to moderate agreement between modalities. Overall discordance ranged from 36% to 50% of trials, and complete directional disagreement (1 modality indicating a decrease, whereas the other indicated an increase) occurred in 11–21% of trials. Inspection of classification proportions revealed systematic differences between modalities. After ASM dose increases, sqEEG more frequently classified seizure frequency as decreased in cases where diaries indicated no change or an increase. Conversely, after ASM dose reductions, sqEEG more frequently classified seizure frequency as increased in cases where diaries indicated no change or a decrease (Figure 2). The categorical analysis remained broadly significant across a range of definitions of the “no change” category, with kappa values remaining in the fair‐to‐moderate range (Supplementary Material 7).

### Absolute Seizure Counts (Generalized Linear Mixed Effects Models)

**Figure 3:**
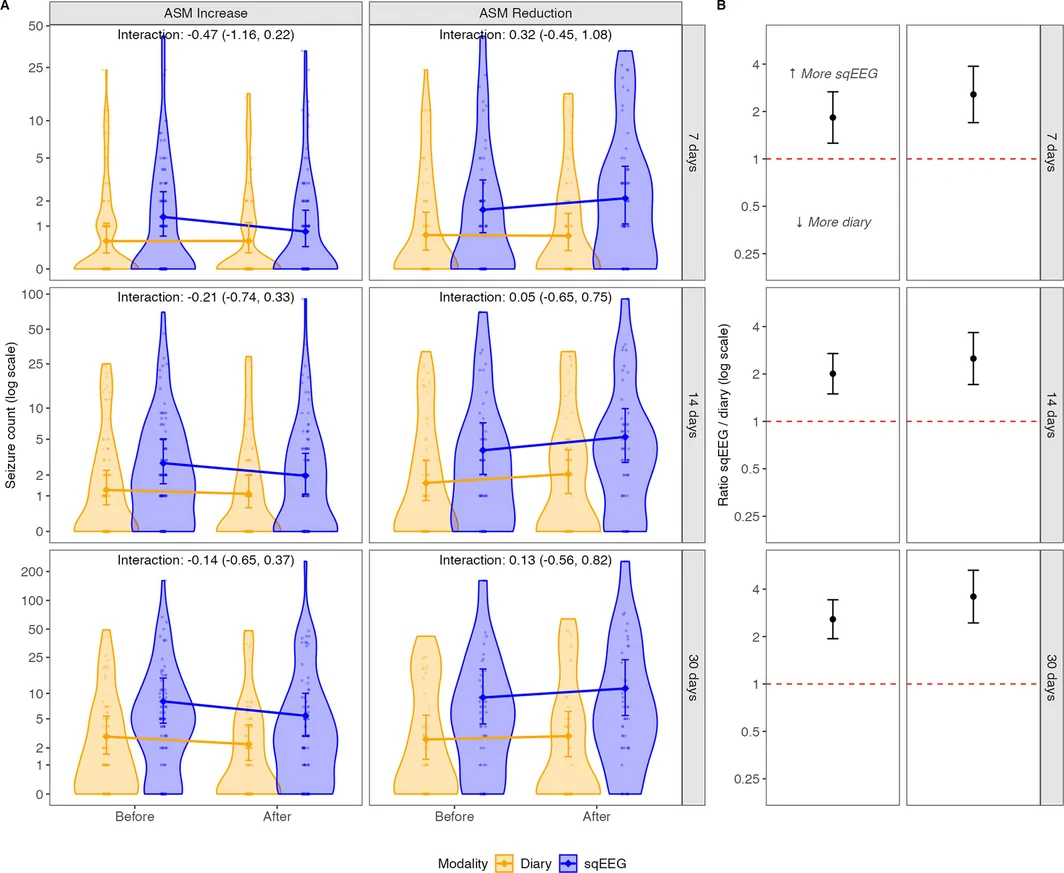
Negative binomial mixed‐effects models of seizure counts before and after antiseizure medication changes, stratified by treatment change type and observation window length. (A) Distribution of observed seizure counts before and after treatment changes by modality (diary – orange; subcutaneous electroencephalogram – blue), displayed on a log scale. Violins show the full distribution of individual trial counts. Diamond‐shaped points and connecting lines represent model‐estimated marginal means with 95% confidence intervals. Interaction terms (modality × time) with 95% confidence intervals, representing the differential pre‐to‐post change between subcutaneous electroencephalogram and diary on the log scale, are displayed within each panel. (B) Estimated ratio of seizure counts detected by subcutaneous electroencephalogram relative to seizure diaries (modality effect), with 95% confidence intervals, displayed on a log scale. A ratio of 1 (dashed line) indicates no difference between modalities. Asterisks indicate statistical significance after Benjamini–Hochberg correction. ASM = antiseizure medication; sqEEG = subcutaneous electroencephalogram. [Color figure can be viewed at www.annalsofneurology.org]

Negative binomial generalized linear mixed‐effects models were used to assess differences in seizure counts between sqEEG and seizure diaries around ASM changes. Across all 6 models (dose increases and reductions analyzed over 7‐, 14‐, and 30‐day windows), there was a significant main effect of modality on seizure counts. In these models, sqEEG recorded significantly more seizures than patient‐reported seizure diaries across all window lengths and change types, with modality effect estimates ranging from 0.78 to 1.21 on the log scale corresponding to 2.2‐ to 3.4‐fold higher counts (Fig. 3B), consistent with the approximately 2‐fold higher total number of sqEEG‐detected seizures compared with diary‐reported events across the cohort (Table 1).

The modality × time interaction did not reach statistical significance in any model (all *p* > 0.05; Fig. 3A, Supplementary Material 13). However, interaction estimates were directionally consistent across all window lengths: after ASM dose increases, sqEEG tended to show larger reductions in seizure counts than diary (interaction estimates on log scale: −0.47, −0.21, −0.14 for 7‐, 14‐, and 30‐day windows; corresponding to 38%, 19%, and 13% larger reductions on the count scale), and after ASM dose reductions, sqEEG tended to show larger increases than diary (interaction estimates: 0.32, 0.05, 0.13; corresponding to 38%, 5%, and 14% larger increases on the count scale). Model diagnostics indicated adequate fit of the negative binomial models, with quantile–quantile plots overall showing no systematic deviations, and DHARMa‐based tests demonstrating no evidence of residual overdispersion, zero inflation, or nonuniformity (Supplementary Material 13). Autocorrelation functions of daily seizure counts remained mostly within the white‐noise significance band across the lag range scanned (5–90 days) for both sqEEG and diary, arguing against systematic bias from seizure cyclicity at the observation window lengths used in the primary analysis (Supplementary Material 9).

### Time to Pre‐Change Seizure Counts (Survival Analysis)

**Figure 4:**
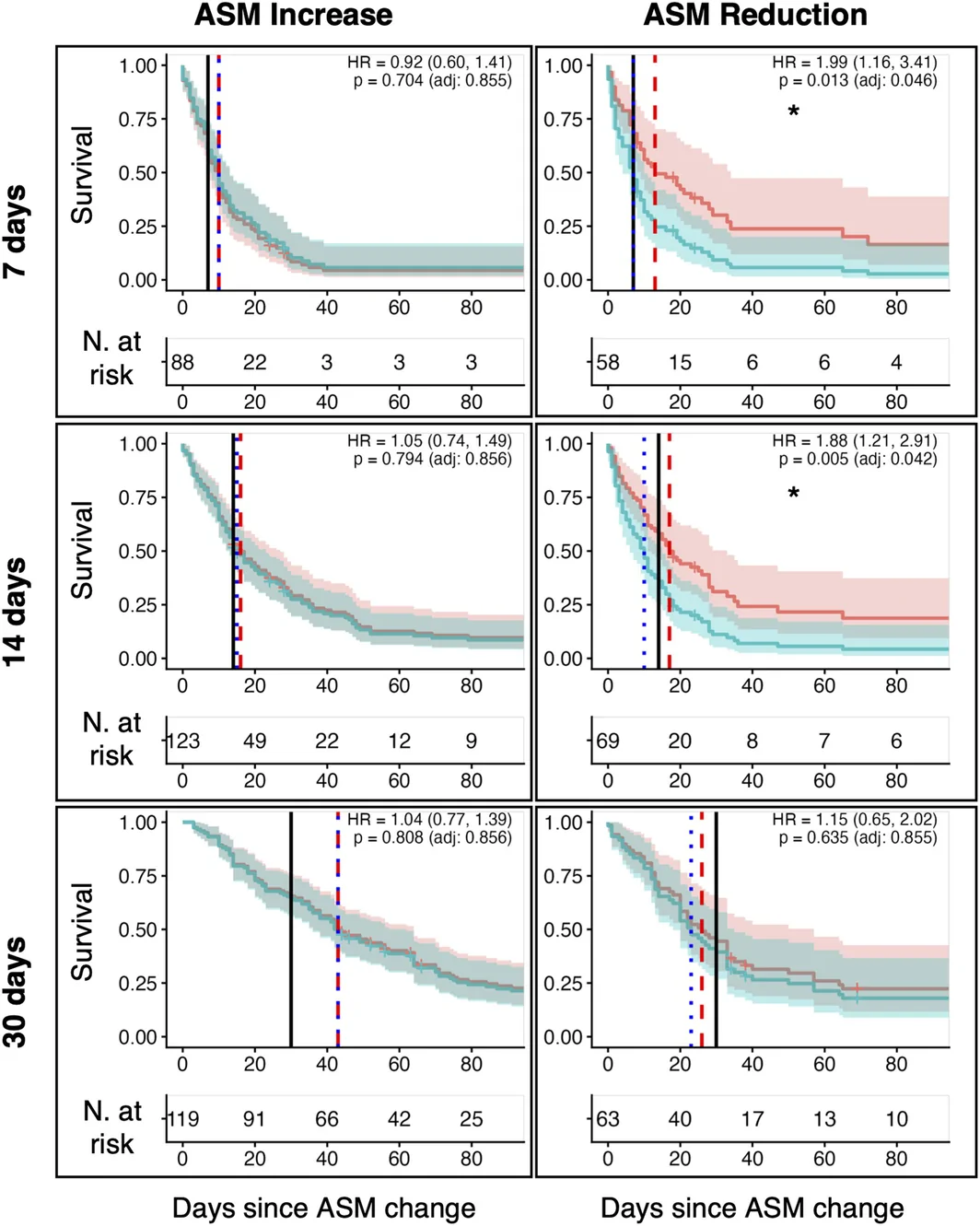
Time to return to baseline seizure count. Model‐adjusted Kaplan–Meier curves are shown for each treatment change category and observation window, stratified by modality (subcutaneous electroencephalogram – blue; diary – orange). The solid black lines indicate the duration of the observation window. Median times to baseline seizure count are indicated by the dotted blue line for subcutaneous electroencephalogram and the dashed orange line for patient‐reported seizure diaries. *Statistically significant effect of modality, corrected for multiple comparisons. ASM = antiseizure medication. [Color figure can be viewed at www.annalsofneurology.org]

We compared the timing of detected changes in seizure frequency between patient‐reported seizure diaries and sqEEG using a survival analysis. The outcome was time to return to the modality‐specific pre‐change seizure count, with modality as a fixed effect. Model‐adjusted Kaplan–Meier survival curves are shown in Fig. 4, with corresponding hazard ratio estimates provided in Supplementary Material 14.

After ASM dose increases, model‐adjusted survival curves for diary and sqEEG data were similar across observation windows. At the 30‐day window, both modalities demonstrated median times to return to baseline seizure count exceeding the window length, indicating delayed return to baseline relative to the observation period.

In contrast, after ASM dose reductions, modality‐dependent differences were observed. At both the 7‐ and 14‐day windows, sqEEG detected return to pre‐change seizure counts significantly earlier than seizure diaries (Benjamini–Hochberg–adjusted p = 0.046 and 0.042, respectively). In the 7‐day window analysis, the median time to baseline seizure count estimated from the model‐adjusted survival curves was 7 days for sqEEG and 13 days for diary data. Similarly, in the 14‐day window analysis, sqEEG detected return to baseline earlier (median 10 days) than diary data (median 17 days). There was no significant difference between modalities at 30 days after ASM dose reductions.

### Sensitivity Analysis

We repeated the 3 analyses restricting to ASM change trials with nonoverlapping observation windows, that is, “pure” changes with no other ASM adjustments within the pre‐ or post‐change periods. Despite the smaller number of trials (see Supplementary Material 1), the results were consistent in direction and magnitude with the primary analyses (see Supplementary Material 15). Categorical outcomes analysis showed a similar distribution of seizure frequency category improvements, despite lack of statistical significance after multiple comparisons corrections (Supplementary Material 15.1). Absolute seizure count models showed a significant effect of modality across all window sizes and treatment change categories, and similar trends in modeled seizure counts (Supplementary Material 15.2). Time to baseline seizure count models also showed a significant difference between modalities in ASM reductions at the 14‐day window (Supplementary Material 15.3).

## Discussion

In this retrospective multicenter cohort study, we evaluated ultra long‐term sqEEG monitoring in patients undergoing antiseizure medication changes across 9 centers. The cohort included >280,000 hours of recorded data and >9,000 sqEEG‐detected seizures. We compared sqEEG recordings with patient‐ or caregiver‐reported seizure diaries and found that sqEEG recorded approximately twice as many seizures as diaries. These findings support the notion that sqEEG might provide a more sensitive and objective measure of seizure frequency, particularly around ASM changes. Our findings extend previous isolated case reports on the usefulness of sqEEG to monitor ASM changes.^15^, ^17^

The low‐to‐moderate and highly variable per‐patient correlations between sqEEG and diary counts are inconsistent with a simple fixed proportional relationship between modalities, suggesting that diary reporting accuracy fluctuates across individuals and over time. The marked difference in time‐of‐day distribution, with sqEEG capturing seizures throughout the 24‐hour period while diary reports clustered during waking hours, highlights the particular potential of sqEEG for detecting nocturnal seizures that patients are less likely to self‐report. The log–log relationship observed for both modalities is consistent with the well‐established L‐relationship in epilepsy seizure count data,^19^ showing that, despite their quantitative differences, they share similar overdispersed statistical properties.

On calibration plots of log‐transformed seizure counts (Supplementary Material 5), sqEEG consistently detected seizures in trials where diary reports were zero, reflected by an offset bias. Regression slopes were positive, but <1, and *R* ^2^ values ranged from 0.11 to 0.40, indicating weak overall association between modalities at the individual trial level. These findings suggest that the relationship between sqEEG and diary seizure counts is not a simple proportional relationship, and that the added clinical value of sqEEG monitoring is likely greatest in patients with low or absent diary‐reported seizure frequency, where the diary by itself provides limited information on whether the underlying condition is improving or worsening.

Our study represents 1 of the largest analyses of simultaneous diary and objective EEG quantification in ambulatory, ultra long‐term settings. Even small ASM dose adjustments produced detectable differences in seizure frequency, as measured by sqEEG, compared with diaries. These observations align with prior studies using gold standard video‐EEG, which demonstrated significant underreporting of seizures by patients or caregivers.^7^, ^12^, ^14^

Categorical analysis of seizure frequency changes (decrease, no change, increase) revealed significant discrepancies between modalities, with discordance in 36 to 50% of trials. In instances of disagreement, sqEEG classifications were more frequently consistent with the expected direction of ASM adjustment (ie, detecting decrease following dose increases and increases after dose reductions), whereas diaries more often indicated no change. These simple categorical measures could be feasibly incorporated into prospective trials of seizure monitoring devices, or clinical practice. Notably, even short observation windows of 7 or 14 days were informative for detecting such changes, suggesting that frequent, short‐term monitoring might be useful for certain clinical scenarios.

Negative binomial generalized linear mixed‐effects models fit the seizure count data well, reflecting the overdispersed nature of seizure frequency and supporting prior work on modeling seizure counts.^24^ Although the modality × time interaction in these models was not statistically significant, sqEEG trends tended to reflect the expected consequences of ASM changes more closely than diaries, particularly over longer observation windows.

Model‐adjusted survival analysis further supported the enhanced sensitivity of sqEEG. Using time to return to baseline seizure count as the outcome, median times derived from Cox model‐adjusted Kaplan–Meier curves were broadly similar for ASM dose increases, likely because each modality's baseline seizure count was used, minimizing the impact of higher absolute sqEEG counts. In contrast, for ASM, dose decreases at the 7‐ and 14‐day windows, sqEEG detected a return to baseline seizure counts significantly earlier than diaries. This discrepancy might reflect a tendency of diaries to underreport breakthrough seizures after dose reduction, which has implications for clinical practice. These findings suggest that relying solely on patient‐reported diaries during ASM tapering might underestimate ongoing seizure activity, and highlight the potential safety benefits of objective monitoring with devices, such as sqEEG, particularly in patients at risk of harm from unrecognized seizures, including sudden unexpected death in epilepsy. This hypothesis warrants further investigation.

ASM increases were less clearly associated with seizure frequency changes compared with ASM reductions, consistent with the literature indicating that in drug‐resistant patients, further dose escalation might have limited efficacy. In newly diagnosed epilepsy, studies show that the majority of patients who achieve seizure freedom do so at lower doses, with incremental dose increases providing modest additional benefit.^28^, ^29^

Several limitations should be considered. The study cohort might not be representative of the broader epilepsy population, as research participants are typically more compliant with study procedures. Research participants have stricter selection criteria and might be better diary reporters, underestimating the real‐world value of sqEEG; in contrast, sqEEG adherence might be lower in nonresearch settings. Reasons for treatment change were not systematically assessed and might have included adverse effects, as well as lack of efficacy. Interictal EEG activity and the impact of ASM changes on seizure cycles were not examined, which should be a focus for future studies.^30^ Timing of medication intake, missed doses, and use of rescue medications were not assessed due to variable schedules across centers. Practices for using sqEEG data to guide clinical decisions varied across centers, ranging from strictly observational studies to clinician‐guided adjustments. Study visits schedules varied between studies. Incomplete adherence to sqEEG recording might have further underestimated seizure frequency changes associated with ASM adjustments. Reasons for nonadherence include removal of the recording device during personal hygiene, but might also include device deficiency (eg, low battery, device malfunction) or device fatigue.^12^

Observation windows in this study were limited to 7, 14, and 30 days. Although standard clinical practice often involves longer follow‐up intervals, longer windows in our dataset would have substantially reduced the number of analyzable trials and increased the likelihood of confounding from additional ASM changes or other clinical factors. Evidence from clinical trials also suggests that medication effects can be observed early during treatment initiation and titration, including for longer‐acting ASMs,^31^, ^32^, ^33^ and this is also supported by responsive neurostimulation device data.^23^

With sqEEG monitoring, semiology assessment is largely limited to the presence or absence of obvious movement‐related artefacts, such as tonic or clonic activity. It is therefore likely that a proportion of electrographic sqEEG seizures represented subclinical seizures, which might have been differentially affected by medication changes. Although the treatment of purely subclinical seizures remains controversial, evidence suggests that many subclinical epileptiform discharges are associated with subtle cognitive symptoms.^34^ In addition, closed‐loop devices, such as responsive neurostimulation, are triggered predominantly by subclinical discharges, yet are associated with reductions in clinical seizure frequency.^35^ We anticipate that understanding the clinical relevance of subclinical seizures will continue to evolve with the availability of large‐scale, real‐world data. Finally, we note that some annotation strategies used across centers, particularly those involving a proportion of manual visual review by neurophysiologists, might not be directly replicable in routine clinical practice. Furthermore, the EpiSight algorithm has known between‐patient variability in detection performance, which might at present limit the generalizability of sqEEG‐based seizure monitoring to patients with clear ictal patterns that are reliably detectable.^14^, ^21^

In conclusion, this study provides evidence that ultra long‐term sqEEG monitoring captures seizure activity more comprehensively than patient‐reported diaries, and might better reflect treatment‐related changes in seizure frequency. These findings support the clinical utility of objective seizure‐monitoring devices, and highlight their potential to improve patient safety and inform treatment decisions in real‐world settings. At present, we believe that sqEEG monitoring is best understood as complementary to patient‐reported diaries; the appropriate weighting of these 2 sources of information in clinical decision‐making warrants prospective investigation. The cost‐effectiveness of sqEEG also remains to be determined and is the topic of ongoing work.^36^

Most of the epilepsy literature, including randomized trials and observational studies, focuses on the initiation of monotherapy or add‐on treatment. Although this approach is appropriate for new‐onset epilepsy and early treatment phases, routine clinical care in refractory epilepsy is dominated by small dose adjustments made in the absence of robust evidence. In this study, we propose an analytical framework specifically designed to evaluate small changes in antiseizure medication doses using outcome measures that better reflect the highly variable, overdispersed nature of seizure counts. This approach aligns more closely with real‐world clinical practice and might facilitate the study of dose optimization strategies in refractory epilepsy. Importantly, such methods could be applied in future real‐world studies and pharmaco‐EEG trials to generate evidence directly relevant to everyday treatment decisions.

## Author Contributions

P.F.V. and M.P.R. contributed to the conception and design of the study; P.F.V., M.M., J.K., C.D., T.L., C.B., K.R., M.T., A.CdC., R.R., L.V., G.R., S.W., C.F‐M., T.W.K., S.B., D.M., K.H., D.S., and R.M. contributed to the acquisition and analysis of data; .PF.V. contributed to drafting the text or preparing the figures.

## Potential Conflicts of Interest

M.P.R. has been a member of ad‐hoc advisory boards for UNEEG medical A/S. P.F.V. received a payment from UNEEG medical A/S for data annotation in an unrelated research study. J.K. received a payment from UNEEG medical AT GmbH for a presentation on subcutaneous EEG at the annual meeting of the Austrian Society for Epilepsy. G.R. has received consultant fees from UNEEG medical A/S for participating in advisory board meeting. R.R. received remuneration from UNEEG medical A/S for a lecture at the 15th EEC. The remaining authors have nothing to report.

## Supporting information


**File S1** 1. Demographic and recording characteristics per center
2. Distribution of seizure counts per time of day
3. L‐Relationship of seizure frequency
4. Correlation between sqEEG and diary counts (full cohort)
5. Calibration plots of seizure counts
6. Number of available trials per observation window
7. Categorical analysis at different definitions of “no change”
8. Distribution of seizure count variables
9. Autocorrelation of seizure counts
10. Number of ASM trials per patient
11. Types of Medication change
12. Categorical Outcomes – Heatmaps
13. Absolute seizure counts – GLMM Negative Binomial Models
14. Time to baseline seizure count models
15. Sensitivity Analysis (non‐overlapping ASM changes)
15.1 Categorical Outcomes
15.2 Absolute seizure counts – GLMM Negative Binomial Models
15.3 Time to baseline seizure count models

## Data Availability

The data that support the findings of this study are available on reasonable request from the corresponding author.
