# Supplementary material for: Monitoring Antiseizure Medication Change Using Ultra Long‐Term Electroencephalogram: A Multicenter Study
Source: Ann Neurol. 2026 Jul 20;100(3):600–12. doi: 10.1002/ana.78305 (PMC13495913; doi:10.1002/ana.78305)
Supplement: Supplementary file 1 [file ANA-100-600-s001.docx]

# Table of Contents

[Table of Contents	1](#_Toc230706768)

[1)	Demographic and recording characteristics per center	3](#_Toc230706769)

[2)	Distribution of seizure counts per time of day	4](#_Toc230706770)

[3)	L-Relationship of seizure frequency	5](#_Toc230706771)

[4)	Correlation between sqEEG and diary counts (full cohort)	6](#_Toc230706772)

[5)	Calibration plots of seizure counts	7](#_Toc230706773)

[6)	Number of available trials per observation window	8](#_Toc230706774)

[7)	Categorical analysis at different definitions of “no change”	9](#_Toc230706775)

[8)	Distribution of seizure count variables	11](#_Toc230706776)

[9)	Autocorrelation of seizure counts	12](#_Toc230706777)

[10)	Number of ASM trials per patient	14](#_Toc230706778)

[11)	Types of Medication change	15](#_Toc230706779)

[12)	Categorical Outcomes - Heatmaps	17](#_Toc230706780)

[13)	Absolute seizure counts - GLMM Negative Binomial Models	20](#_Toc230706781)

[14)	Time to baseline seizure count models	22](#_Toc230706782)

[15)	Sensitivity Analysis (non-overlapping ASM changes)	23](#_Toc230706783)

[15.1) Categorical Outcomes	23](#_Toc230706784)

[15.2) Absolute seizure counts - GLMM Negative Binomial Models	24](#_Toc230706785)

[15.3) Time to baseline seizure count models	27](#_Toc230706786)

# 1) Demographic and recording characteristics per center

| **Anonymised centre ID^#^** | **N. of subjects** | **Age, median (range)** | **N. of ASM trials Increase / Reduction** | **Study duration, median days (range)** | **sqEEG adherence, median (range)** | **Annotation methodology** | **Estimated sensitivity vs. full sqEEG visual review*** |
| --- | --- | --- | --- | --- | --- | --- | --- |
| 1 | 11 | 36 (26-63) | 11 / 8 | 433 (45-538) | 78.4% (23.1-98.6%) | Expert review of Episight + diary timestamps + 10% random review; full review if sensitivity <80% | >80% |
| 2 | 10 | 36.5 (21-55) | 32 / 21 | 207.5 (112-447) | 76.7% (56.1-94.4%) | Expert review of Episight | 70.5-91.5%^$^ |
| 3 | 7 | 37 (29-54) | 2 / 0 | 174 (62-195) | 81.7% (66.1-96.4%) | Expert review of Episight | 70.5-91.5%^$^ |
| 4 | 8 | 30 (27-59) | 5 / 2 | 230 (155-459) | 87.7% (66.2-98.3%) | Expert review of Episight | 70.5-91.5%^$^ |
| 5 | 5 | 41 (21-45) | 0 / 0 | 82 (83-84) | 90.7% (78.9-99.1%) | Expert review of Episight | 70.5-91.5%^$^ |
| 6 | 4 | 55 (52-71) | 8 / 3 | 160.5 (150-177) | 78.4% (23.1-98.6%) | Expert review of Episight + full review (two independent raters) | 100% |
| 7 | 5 | 41 (34-53) | 21 / 8 | 294 (67-385) | 92.7% (72.4-93.2%) | Expert review of Episight + diary timestamps + aEEG of first two weeks | 70.5-91.5%^$^ |
| 8 | 5 | 41 (31-52) | 11 / 11 | 535 (40-910) | 84.2% (39.3-94.9%) | Expert review of Episight + diary timestamps | 70.5-91.5%^$^ |
| 9 | 2 | 40 (23-57) | 0 / 1 | 244.5 (149-340) | 60.8% (48.9-72.7%) | Expert review of Episight + diary timestamps + full review of at least two randomly selected days | 70.5-91.5%^$^ |
| 10 | 8 | 44 (27-75) | 11 / 5 | 81 (69-94) | 73.4% (44.1-91.2%) | Expert review of Episight + Full review (three independent raters) | 100% |

^#^one center conducted two separate studies, with different protocols, hence they are differentially described.

*the ground truth is defined as full review of the sqEEG dataset by at least one experienced neurophysiologist

$ based on median per patient sensitvity of Episight comared to visual review, from three references (Viana et al, Rubboli et al, Remvig et al)

# 2) Distribution of seizure counts per time of day

# 3) L-Relationship of seizure frequency

# 4) Correlation between sqEEG and diary counts (full cohort)

Each datapoint represents a patient. Each patient’s data was binned into 7, 14 or 30-day bins. Patients with at least 4 full bins included.

# 5) Calibration plots of seizure counts

# 6) Number of available trials per observation window

|  | Number of complete / non-overlapping observation windows | | |
| --- | --- | --- | --- |
|  | 7 days | 14 days | 30 days |
| ASM Increase | 101 / 80 | 99 / 45 | 82 / 23 |
| ASM Decrease | 59 / 50 | 56 / 30 | 45 / 13 |

# 7) Categorical analysis at different definitions of “no change”

**Sensitivity of the categorical analysis to the definition of "no change."** Categorical analysis recomputed across tolerance bands of ±k seizures (k = 0 to 5), where any difference in seizure count between pre- and post-change windows within ±k was reclassified as "no change". (A) Raw Stuart–Maxwell p-value for marginal homogeneity as a function of tolerance band; the horizontal dotted line indicates p = 0.05. (B) Overall percentage of trials with discordant categorical classification between modalities. (C) Linearly weighted Cohen's kappa. (D) Percentage of trials classified as "no change" by each modality (sqEEG, blue; Diary, orange) as a function of tolerance band; solid lines denote ASM increases and dashed lines ASM reductions.

# 8) Distribution of seizure count variables

# 9) Autocorrelation of seizure counts

**Cross-patient autocorrelation function (ACF) of daily seizure counts across lags 5 to 90 days.** The autocorrelation function was computed per patient on daily sqEEG and diary seizure counts, across lags from 5 days to one quarter of each patient's recording length (capped at 90 days). Solid lines show the cross-patient median ACF at each lag; shaded patches show the interquartile range. Dashed horizontal lines indicate the median Bartlett 95% white-noise significance band across patients.

**Distribution of the strongest significant ACF peak per patient.** For each patient and modality, the lag at which the most extreme significant ACF value occurred (Bartlett 95% white-noise band) was recorded as that patient's strongest peak. Patients with no significant ACF value at any scanned lag are not represented. Bars show the percentage of patients per modality whose strongest significant peak fell within 5-day lag bins. Significant peaks include both positive and negative ACF values.

# 10) Number of ASM trials per patient

Distribution of number of ASM trials per patient.

# 11) Types of Medication change

Total number of ASM change trials with complete observation windows (shortest 7-day window)

Total number of ASM trials with non-overlapping windows (shortest 7-day window)

# 12) Categorical Outcomes - Heatmaps

# 13) Absolute seizure counts - GLMM Negative Binomial Models

Model Fit

| **Change Type** | **Window** | **AIC** | **BIC** | **Log-Likelihood** | **Overdispersion p-value** | **Zero Inflation p-value** | **Uniformity p-value** | **Random Variances** |
| --- | --- | --- | --- | --- | --- | --- | --- | --- |
| Decrease | 7 | 877 | 898 | -433 | 0.972 | 0.848 | 0.490 | 1.783 |
| Decrease | 14 | 1091 | 1111 | -539 | 0.978 | 0.726 | 0.638 | 1.421 |
| Decrease | 30 | 1045 | 1064 | -516 | 0.996 | 0.254 | 0.310 | 1.903 |
| Increase | 7 | 1181 | 1205 | -584 | 0.710 | 0.928 | 0.196 | 2.065 |
| Increase | 14 | 1565 | 1589 | -776 | 0.536 | 0.986 | 0.341 | 2.168 |
| Increase | 30 | 1642 | 1665 | -815 | 0.514 | 0.380 | 0.022 | 2.174 |

p < 0.05 in bold.

Model Coefficients

| **Change Type** | **Window** | **Time*** | **p-value** | **Modality^+^** | **p-value** | **Time X Modality** | **p-value** | **p-value (adj.)** |
| --- | --- | --- | --- | --- | --- | --- | --- | --- |
| Decrease | 7 | -0.029  (-0.602, 0.544) | 0.921 | 0.784  (0.221, 1.347) | 0.006 | 0.317  (-0.45, 1.083) | 0.418 | 0.734 |
| Decrease | 14 | 0.271  (-0.247, 0.789) | 0.305 | 0.896  (0.371, 1.421) | 0.001 | 0.048  (-0.654, 0.749) | 0.894 | 0.894 |
| Decrease | 30 | 0.108  (-0.401, 0.616) | 0.679 | 1.212  (0.685, 1.739) | <0.001 | 0.13  (-0.562, 0.822) | 0.713 | 0.855 |
| Increase | 7 | 0.009  (-0.505, 0.524) | 0.971 | 0.841  (0.343, 1.34) | 0.001 | -0.473  (-1.163, 0.218) | 0.180 | 0.360 |
| Increase | 14 | -0.145  (-0.535, 0.246) | 0.467 | 0.801  (0.407, 1.194) | <0.001 | -0.206  (-0.739, 0.327) | 0.449 | 0.734 |
| Increase | 30 | -0.253  (-0.63, 0.125) | 0.189 | 1.017  (0.639, 1.395) | <0.001 | -0.141  (-0.649, 0.366) | 0.585 | 0.855 |

* time (pre to post) coefficient for the baseline modality (diary)

^+^ diary as baseline

QQ residuals plots

# 14) Time to baseline seizure count models

| **Change Type** | **Window** | **HR** | **95% CI (lower)** | **95% CI (upper)** | **p-value** | **p-value (adj.)** |
| --- | --- | --- | --- | --- | --- | --- |
| Increase | 7 | 0.920 | 0.599 | 1.413 | 0.704 | 0.855 |
| Increase | 14 | 1.048 | 0.737 | 1.490 | 0.794 | 0.856 |
| Increase | 30 | 1.037 | 0.771 | 1.395 | 0.808 | 0.856 |
| Decrease | 7 | 1.986 | 1.157 | 3.410 | 0.013 | **0.046** |
| Decrease | 14 | 1.879 | 1.214 | 2.908 | 0.005 | **0.042** |
| Decrease | 30 | 1.147 | 0.651 | 2.022 | 0.635 | 0.855 |

p < 0.05 in bold.

# 15) Sensitivity Analysis (non-overlapping ASM changes)

## 15.1) Categorical Outcomes

## 15.2) Absolute seizure counts - GLMM Negative Binomial Models

Model fit

| **Change Type** | **Window** | **AIC** | **BIC** | **LogLik** | **Overdispersion p-value** | **ZeroInflation p-value** | **Uniformity p-value** | **Random Variances** |
| --- | --- | --- | --- | --- | --- | --- | --- | --- |
| Increase | 7 | 928 | 951 | -458 | 0.604 | 1 | 0.876 | 1.96 |
| Decrease | 7 | 691 | 711 | -340 | 0.988 | 0.778 | 0.406 | 2.32 |
| Increase | 14 | 746 | 765 | -367 | 0.932 | 0.97 | 0.643 | 2.01 |
| Decrease | 14 | 511 | 527 | -249 | 0.668 | 0.99 | 0.773 | 2.63 |
| Increase | 30 | 447 | 462 | -218 | 0.692 | 0.89 | 0.064 | 3.14 |
| Decrease | 30 | 273 | 285 | -131 | 0.428 | 0.876 | 0.81 | 3.55 |

Model coefficients

| **Change**  **Type** | **Window** | **Time** | **p-value** | **Modality** | **p-value** | **Time X Modality** | **p-value** | **p-value (adj.)** |
| --- | --- | --- | --- | --- | --- | --- | --- | --- |
| Increase | 7 | -0.122  (-0.679, 0.434) | 0.667 | 0.811  (0.281, 1.341) | 0.003 | -0.471  (-1.221, 0.28) | 0.219 | 0.493 |
| Decrease | 7 | -0.078  (-0.733, 0.578) | 0.817 | 1.013  (0.383, 1.643) | 0.002 | 0.204  (-0.662, 1.07) | 0.644 | 0.825 |
| Increase | 14 | -0.241  (-0.767, 0.285) | 0.369 | 0.874  (0.354, 1.393) | 0.001 | -0.147  (-0.861, 0.568) | 0.688 | 0.825 |
| Decrease | 14 | 0.031  (-0.723, 0.786) | 0.935 | 0.889  (0.165, 1.613) | 0.016 | 0.304  (-0.702, 1.31) | 0.553 | 0.825 |
| Increase | 30 | -0.348  (-0.942, 0.246) | 0.251 | 0.669  (0.094, 1.245) | 0.023 | -0.18  (-1.001, 0.642) | 0.668 | 0.825 |
| Decrease | 30 | 0.109  (-1.056, 1.274) | 0.855 | 1.672  (0.66, 2.684) | 0.001 | 0.119  (-1.334, 1.573) | 0.872 | 0.880 |

QQ residual plots

## 15.3) Time to baseline seizure count models

| **Change Type** | **Window** | **HR** | **95% CI (lower)** | **95% CI (upper)** | **p-value** | **p-value (adj.)** |
| --- | --- | --- | --- | --- | --- | --- |
| Increase | 7 | 0.851 | 0.607 | 1.193 | 0.349 | 0.629 |
| Increase | 14 | 1.048 | 0.573 | 1.917 | 0.880 | 0.880 |
| Increase | 30 | 0.920 | 0.407 | 2.076 | 0.840 | 0.880 |
| Decrease | 7 | 1.848 | 1.160 | 2.944 | 0.010 | 0.087 |
| Decrease | 14 | 2.487 | 1.441 | 4.290 | 0.001 | **0.019** |
| Decrease | 30 | 1.724 | 0.417 | 7.131 | 0.452 | 0.740 |

p < 0.05 in bold.
